# Continuous lighting can improve yield and reduce energy costs while increasing or maintaining nutritional contents of microgreens

**DOI:** 10.3389/fpls.2022.983222

**Published:** 2022-09-30

**Authors:** Jason Lanoue, Sarah St. Louis, Celeste Little, Xiuming Hao

**Affiliations:** Harrow Research and Development Centre, Agriculture & Agri-Food Canada, Harrow, ON, Canada

**Keywords:** continuous lighting, microgreens, antioxidant, phenolics, anthocyanin, energy efficiency, plant factory with artificial light, indoor vertical farming

## Abstract

Microgreens represent a fast growing segment of the edible greens industry. They are prized for their colour, texture, and flavour. Compared to their mature counterparts, microgreens have much higher antioxidant and nutrient content categorizing them as a functional food. However, current production practices in plant factories with artificial light are energy intensive. Specifically, the lack of sunlight within the indoor structure means all of the light must be provided *via* energy consuming light fixtures, which is energy intensive and costly. Plant growth is usually increased with the total amount of light provided to the plants - daily light integral (DLI). Long photoperiods of low intensity lighting (greater than 18h) providing the desired/target DLI can reduce the capital costs for light fixtures and electricity costs. This is achieved by moving the electricity use from peak daytime hours (high price) to off-peak hours (low price) during the night in regions with time-based pricing scheme and lowering the electricity use for air conditioning, if plant growth is not compromised. However, lighting with photoperiods longer than tolerance thresholds (species/cultivar specific) usually leads to plant stress/damage. Therefore, we investigated the effects of continuous 24h white light (CL) at two DLIs (~14 and 21 mol m^-2^ d^-1^) on plant growth, yield, and antioxidant content on 4 types of microgreens - amaranth, collard greens, green basil, and purple basil to see if it compromises microgreen production. It was found that amaranth and green basil had larger fresh biomass when grown under CL compared to 16h when the DLIs were the same. In addition, purple basil had higher biomass at higher DLI, but was unaffected by photoperiods. Plants grown under the CL treatments had higher energy-use-efficiencies for lighting (10-42%) than plants grown under the 16h photoperiods at the same DLI. Notably, the electricity cost per unit of fresh biomass ($ g^-1^) was reduced (8-38%) in all microgreens studied when plants were grown under CL lighting at the same DLIs. Amaranth and collard greens also had higher antioxidant content. Taken together, growing microgreens under CL can reduce electricity costs and increase yield while maintaining or improving nutritional content.

## 1 Introduction

Microgreens are a fast growing specialty crop within the edible greens industry (Kyriacou et al., 2016). They are prized for their colour, texture, and flavour. Microgreens are typically harvested within 3 weeks of sowing and can be harvested before or at the first true leaf stage depending on desired use. Although smaller in size, microgreens have higher nutritional content than their mature counterparts, and thus are considered a functional food (Xiao et al., 2012; Choe et al., 2018; Kyriacou et al., 2019).

Due to their small size and compact growing strategy, microgreens are typically grown in indoor vertical farms or plant factories with artificial light to maximize yield per unit of land area (Graamans et al., 2018). The terms indoor vertical farm and plant factory are typically used synonymously but usage varies based on geographical location. Generally, indoor vertical farm is used in North America whereas plant factories with artificial light (PFAL) is used in Europe and Asia. Both refer to the use of multi-layer growing platforms (i.e., vertical farming) inside warehouses or insulated shipping containers for production with artificial light as the sole light source (Kozai and Niu, 2016). Throughout this manuscript we will use the term plant factory. While this type of growing system can have very high yield per unit of land area, it is energy intensive. All the light required for plant growth and photosynthesis needs to come from artificial lighting with the use of electricity (Shibaeva et al., 2022b). Even if the adoption of the energy-efficient light-emitting diode (LED) fixtures can reduce the electricity use (van Delden et al., 2021), the electricity used by LED lighting still represents upwards of 20% of operating costs in plant factories; second only to labour (Kozai and Niu, 2019). Furthermore, this type of growing system is also capital intensive. It not only uses expensive LED lighting systems (even though their price has come down) but also uses heating, ventilation, and air conditioning (HVAC) to control temperature and humidity. The majority of input electricity to the lighting system will eventually becoming heat since plants typically only convert 1-5% of the incoming radiation to biomass (Zhu et al., 2008; Kozai and Niu, 2019). The excessive heat load and the high humidity from plant transpiration requires its removal and dehumidification *via* air conditioning systems to maintain an optimal growing environment for the plants, which, in addition to lighting, increases electricity costs (Goto, 2012; Kozai and Niu, 2016). These high input costs are in part why only 50% of plant factories in Japan were profitable in 2018 (Kozai and Niu, 2019), and why plant factories struggle to be used as a mainstream producer of edible greens elsewhere in the world (Kozai, 2018). Based on the poll conducted by Indoor AgTech Innovation Virtual Summit 2021 (https://indooragcenter.org/indoor-agtech-virtual-summit-2021/), high energy cost is the main limiting factor for profitable production with plant factories. Therefore, innovation in lighting systems and strategies is the key to reduce energy costs and improve energy efficiency.

Plant growth and yield are usually determined by the amount of light intercepted by the plant during a day - daily light integral (DLI; photosynthetic photon flux density (PPFD) x photoperiod duration). Both the increase in PPFD (Samuoliene et al., 2013) and an extension in photoperiod (Demers and Gosselin, 2002) can increase DLIs and have been shown to increase biomass production up to a saturation point. With respect to PPFD, beyond a certain species-specific limit, no further increase in biomass is observed and further increases to the PPFD can be detrimental to the plant (Demmig-Adams and Adams, 1992; Szymańska et al., 2017) since as PPFD increases, the quantum yield (i.e., the increase in CO_2_ fixed per additional photon) decreases (Lanoue et al., 2017). Furthermore, the use of high PPFD can increase the transpiration rate of plants, exacerbating the aforementioned humidity issue (Goto, 2012; Lanoue et al., 2018).

Similar to an increase in PPFD, photoperiod extension can be used to increase DLI and biomass (Demers and Gosselin, 2002). The ultimate goal in photoperiod extension is 24h lighting/continuous lighting (CL). It is more economical to use long photoperiods (18h up to 24h) of low PPFD (<200 µmol m^-2^ s^-1^) to achieve the target/desired DLIs because it reduces the capital cost of light fixtures (Hao et al., 2018). The longer the photoperiod, the lower the PPFD that can be used to reach the desired DLI. It should be noted that the DLI requirements vary between plant species and cultivars. Therefore the definition of a long photoperiod, low PPFD lighting strategy will be species-specific.

In many regions of the world which employ time-of-use pricing (TOUP) such as Ontario, Canada, some states in the USA, 17 European countries including France, Sweden, Germany, Finland, as well as South Korea, the price of electricity is much higher in the peak hours during daytime (when demand is highest) compared to the price in the off-peak hours during the night (IRENA, 2019; IESO, 2022). It is important to note that the form in which TOUP is utilized in each country may be different (i.e., static time-of-use pricing, real-time pricing, variable peak pricing, or critical peak pricing), but regardless of strategy, off peak pricing is always cheaper than on peak. Therefore, long photoperiod, low PPFD lighting such as CL can also reduce electricity costs by moving part of electricity use from daytime to nighttime when prices are at their lowest in these regions (Hao et al., 2018; IESO, 2022). At lower PPFD, both the heat load from lighting and plant transpiration decrease, reducing the usage of electricity by the air conditioning system to remove heat and moisture to maintain optimal growing environment for plants (Kozai and Niu, 2016).

The use of long photoperiod lighting such as 24h CL means constant photon energy is provided to the plant allowing for 24h CO_2_ fixation and growth. In this way, it has been hypothesized that the use of CL can increase plant production (Sysoeva et al., 2010; Velez-Ramirez et al., 2011; Velez-Ramirez et al., 2012; Shibaeva et al., 2022a). However, exceeding the tolerable photoperiod limits, which are species-specific, can lead to diminished yield, photoperiod-related leaf injury, and an economic disadvantage for growers (Demers and Gosselin, 2002; Hao et al., 2018). Some plant species such as tomato and pepper have reduced yield and leaf injury characterized by chlorosis when grown under CL (Murage and Masuda, 1997; Velez-Ramirez et al., 2017). It is hypothesized that CL-injury is due to a mismatch between environmental cues and endogenous circadian rhythms (Velez-Ramirez et al., 2017; Marie et al., 2022). Specifically, since the plant is under constant light, it seems that components of the light harvesting complex are negatively affected causing reduced transcription leading to inadequate use and/or dissipation of light (Velez-Ramirez et al., 2014). More recent research in these two crops have provided evidence that dynamic CL, which involves a change in light spectrum between daytime and nighttime, can result in injury-free production (Lanoue et al., 2019; Lanoue et al., 2022). Some cultivars of lettuces and some members of the Brassicaceae microgreen family have also been shown to have positive interactions with CL (Ohtake et al., 2018; Shibaeva et al., 2022b). Since the production period of microgreens is short, and CL-injury in tomatoes and peppers tends to take more than a month to have noticeable reductions in yield (Lanoue et al., 2021a), we hypothesize that CL may not compromise the production of microgreens and could be a viable production strategy.

However, CL can increase harmful reactive oxygen species (ROS) due to the stress from constant light exposure to the plant (Haque et al., 2015; Huang et al., 2019). Subsequently, the concentration of ROS scavenging molecules such as antioxidants can also be increased during CL (Haque et al., 2015). The extent of injury is largely linked to the interplay between ROS production and scavenging ability. If this homeostatic balance becomes too heavily skewed by elevated ROS production, then leaf injury will occur, resulting in detrimental plant growth and yield. However, if one can balance the oxidative pressure with the antioxidant synthesis, injury-free production is feasible. Therefore, the prospect of injury-free production under CL coupled with the hormetic impact can increase antioxidants/health promoting compounds in plants; which is an intriguing possibility for microgreen production in plant factories.

As such, we studied the impact of PPFD and photoperiod (including CL) on plant growth, yield and nutritional content of 4 types of microgreens in order to assess if long photoperiod (CL) and low intensity lighting can be used to improve the sustainability/energy efficiency in indoor production of microgreens grown in plant factories.

## 2 Materials and methods

### 2.1 Plant materials and lighting treatments

Four types of microgreens were used in the study. Two hundred seeds each of amaranth (*Amaranthus tricolor*) cv. ‘Garnet Red’, collard greens (*Brassica oleracea* var. *viridis*) cv. ‘Vates’, as well as basil (*Ocimum basilicum*) cv. ‘Genovese’ and cv. ‘Red Rubin’ (henceforth referred to as green and purple basil respectively; Johnny’s Select Seeds, Fairfield, Maine, USA) were sown into individual trays filled with Berger BM6 All-Purpose potting soil (Berger, Saint-Modeste, Quebec, Canada). Once sown, the trays were placed in a germination chamber at Agriculture & Agri-Food Canada’s Harrow Research and Development Centre with a constant temperature of 24°C and a relative humidity of 90% in complete darkness. Amaranth and collard greens remained in the chamber for 3 days while basil was in the germination chamber for 5 days. Upon germination, trays of each cultivar were placed into four different growth areas (1.93 m^2^) within Conviron walk-in growth chambers (PGW40; Conviron, Winnipeg, MB, Canada) each containing one of four lighting treatments (**Table 1**). The growth chamber temperature was maintained at 22°C (24 hour) while the relative humidity was kept between 60-70%. Plants were irrigated as needed. Harvest occurred 11 days after sowing for amaranth and collard greens and 19 days after sowing for both basil cultivars.

**Table 1:** Light treatments provided by the Flexstar 645W dimmable LED fixtures (Flexstar, California, USA) in Conviron walk-in growth chambers (PGW40; Conviron, Winnipeg, MB, Canada) measured at the height of the top of the tray in 6 different locations within the chamber.

Treatment (DLI/photoperiod)	PPFD (µmol m^-2^ s^-1^)	DLI (mol m^-2^ d^-1^)	Photoperiod (h)
14DLI/16h	250.8 ± 2.1	14.5 ± 0.1	16
14DLI/24h	166.6 ± 2.7	14.4 ± 0.2	24
21DLI/16h	376.8 ± 1.7	21.7 ± 0.1	16
21DLI/24h	247.6 ± 7.9	21.4 ± 0.7	24

**Figure 1:**
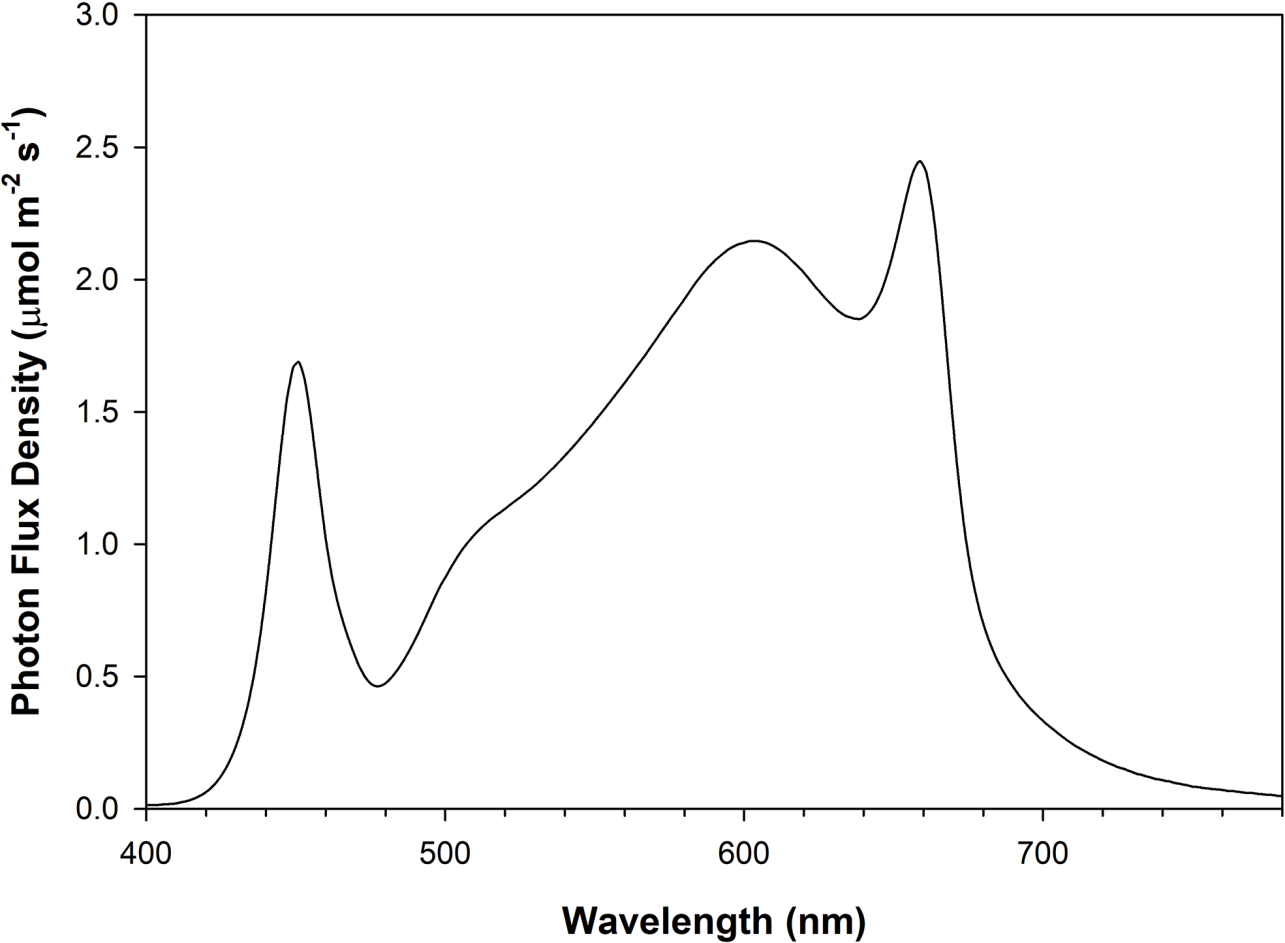
Photon flux density (PFD) distribution of Flexstar 645W dimmable LED fixtures (Flexstar, California, USA) measured using a Li-180 spectrometer (Li-COR Biosciences Inc. Lincoln, Nebraska, USA).

The four light treatments consisted of 2 levels of DLIs (14 and 21 mol m^-2^ day^-1^) and 2 photoperiods (16h and 24h; **Table 1**). Throughout the manuscript, the lighting treatments will be represented using the following notation: DLI/photoperiod. Lighting treatments were chosen based on similar PPFD and photoperiods in other studies where between 200-300 µmol m^-2^ s^-1^ was the typical PPFD used with 16h photoperiods (Samuoliene et al., 2013; Viršilė et al., 2019; Pennisi et al., 2020; Shibaeva et al., 2022b; Sutulienė et al., 2022; Vaštakaitė-Kairienė et al., 2022). Using that as a baseline for the 14DLI/16h treatment, the other treatments we determined by controlling either DLI but extending the photoperiod or controlling the PPFD and extending the photoperiod. All light treatments were provided by Flexstar 645W dimmable LED fixtures (Flexstar, California, USA) and were the same broad/white spectrum (**Figure 1**). The growth chamber trials were replicated 3 times in 2022.

### 2.2 Growth measurements

The plants were harvested by cutting them at the junction where their base meets the growth media. Plant height was measured on five random plant samples from each treatment of each cultivar during each replicate (i.e., 15 total samples per microgreen per treatment). Total fresh weight was obtained then a subsample was flash frozen in liquid nitrogen and placed in a -80°C freezer until analysis. Another subsample was weighed, then placed in a 70°C oven for 1 week then re-weighed to obtain the dry matter percentage of the sample. Energy-use-efficiency of the lights (EUEL) only was calculated using the total fresh biomass obtained and dividing it by the cumulative input of energy into the lighting fixtures during the production period (g FW MJ^-1^).

### 2.3 Photosynthetic pigment analysis

Frozen tissue was lyophilized then ground. One mL of 95% ethanol was then added to the sample and the tube was placed in a water bath at 50°C for 3 hours. The tube was centrifuged at 13000 rpm for 1 minute before the supernatant was removed and placed in a clean tube. The process was repeated and both aliquots were combined for a total extract volume of 2 mL. Samples were then analyzed at 664 nm, 649 nm, and 470 nm in a UV/VIS spectrophotometer (UV-1600PC. VWR. Mississauga, Ontario, Canada). Concentrations of chlorophyll *a*, *b*, and carotenoids were determined using the equations from Lichtenthaler (1987).

### 2.4 Antioxidant assays

#### 2.4.1 DPPH (2,2-diphenyl-1-picrylhydrazyl) assay

The antiradical activity in microgreen tissue was determined based on a modified version of a previously reported method (Alrifai et al., 2020). Tissue samples that were previously frozen in liquid nitrogen and stored in a -80°C freezer were removed and lyophilized. Lyophilized tissue was ground in a homogenizer then 1 mL of 100% methanol was added to the microfuge tube. The sample was then left on a nutator overnight at room temperature. The next morning, the samples were centrifuged at 13,000 rpm for 5 minutes. The supernatant was collected in a clean tube before re-suspending the pellet in 1 mL of fresh 100% methanol. The sample was placed on a nutator for 3 hours before being centrifuged and having the supernatant removed. Both supernatant fractions were mixed in a single tube and placed in -20°C freezer until analysis. Fresh 2,2-diphenyl-1-picrylhydrazyl (DDPH; 350 µM) was prepared immediately before analysis. In a cuvette, 1 mL of DPPH was mixed with 125 µL of sample and placed in the dark to incubate for 30 minutes before the absorbance was measured at 517 nm. This procedure was completed in duplicate. A standard curve was completed in triplicate using the same assay technique with ascorbic acid used in place of the tissue sample.

#### 2.4.2 FRAP (ferric reducing antioxidant power) assay

The ferric reducing antioxidant power (FRAP) assay of microgreen tissue was determined using a modified version of a previously reported method (Alrifai et al., 2020). Samples were extracted using a method similar to the DPPH analysis. FRAP reagent was made at the time of analysis and consisted of 300 mM acetate buffer (pH 3.6), 20 mM FeCl_3_, and 10 mM 2, 4, 6-Tris (2-pyridyl)-s-triazine (TPTZ) in 40 mM HCl. 100 µL of methanolic sample extract was mixed with 900 µL of FRAP reagent and incubated at 37°C for 2h before reading the absorbance at 593 nm. A standard curve was completed using the same assay technique with ascorbic acid used in place of the tissue sample.

#### 2.4.3 Total phenolic content

Total phenolic content was determined using a modified protocol from Ainsworth and Gillespie (2007). Briefly, 100 µL of methanolic sample extract was combined with 200 µL of Folin-Ciocalteu’s reagent (Thermo Fisher Scientific, MA, USA) and 800 µL of 700 mM sodium carbonate. The tubes were vortexed for 30 seconds then allowed to stand at room temperature for 2h. The absorbance was measured at 765 nm using a UV/VIS spectrophotometer. Total phenolic content was expressed as gallic acid equivalents.

#### 2.4.4 Total anthocyanin

Determination of total anthocyanin content was done using a slightly modified protocol from Lee et al. (2005). The assay began by adding 100 µL of methanolic sample to both 1 mL of potassium chloride (0.025 M; pH = 1.0) and 1 mL of sodium acetate (0.4 M; pH = 4.5) separately. The mixtures were incubated at room temperature for 30 minutes before the absorbance was measured at both 520 nm and 700 nm. Anthocyanin contents were then calculated using the following equation:


$$Anthocyanin\;content=\frac{\left(A*MW*DF*10^{3}\right)}{\epsilon *l}$$

Where *A* is the absorbance (*A*=(*A*_520nm_-*A*_700nm_)_pH1.0_ – (*A*_520nm_-*A*_700nm_)_pH4.5_), *MW* is the molecular weight of cyanidin-3-glucoside (449.2 gmol^-1^), *DF* was the dilution factor, 10^3^ is the factor to convert g to mg, *ϵ*is the molar extinction coefficient of cyanidin-3-glucoside (26900 L mol^-1^) and *l* is the path length of 1 cm.

### 2.5 Electricity Cost Calculation

The electricity cost ($ g^-1^ FW) from LED lighting only during each production period of all microgreens was calculated using the following equation:


$$Electicity\;cost=\frac{\left[\sum_{n=0}^{n}\left(\left(\frac{Lu}{Lm}\right)\left(\frac{P}{10^{6}}\right)\right)E_{n}\right]}{FW}$$

Where *n* is the hour, *Lu* is the PPFD used, *Lm* is the maximum PPFD of the fixture, *P* is the input wattage of the fixture (W), 10^6^ is a conversion factor from W to MW, *E_n_* is the electricity price at a given hour (*n*) as determined from IESO, 2022, and *FW* is the fresh weight (g) produced for a given microgreen during a specific production period. Fresh weight was a measure of total biomass produced by each microgreen under each light treatment at the end of the growth period.

### 2.6 Statistics

For each microgreen, the experiment was replicated 3 times. For each of the pigment analyses and antioxidant analyses, 2 subsamples were taken from each destructive harvest. All statistics were performed using SAS studio 3.5. A two-way ANOVA was performed and a multiple means comparison was done using a Tukey-Kramer adjustment with a p<0.05 indicating a significant difference.

## 3 Results

### 3.1 Plant growth and yield

**Figure 2:**
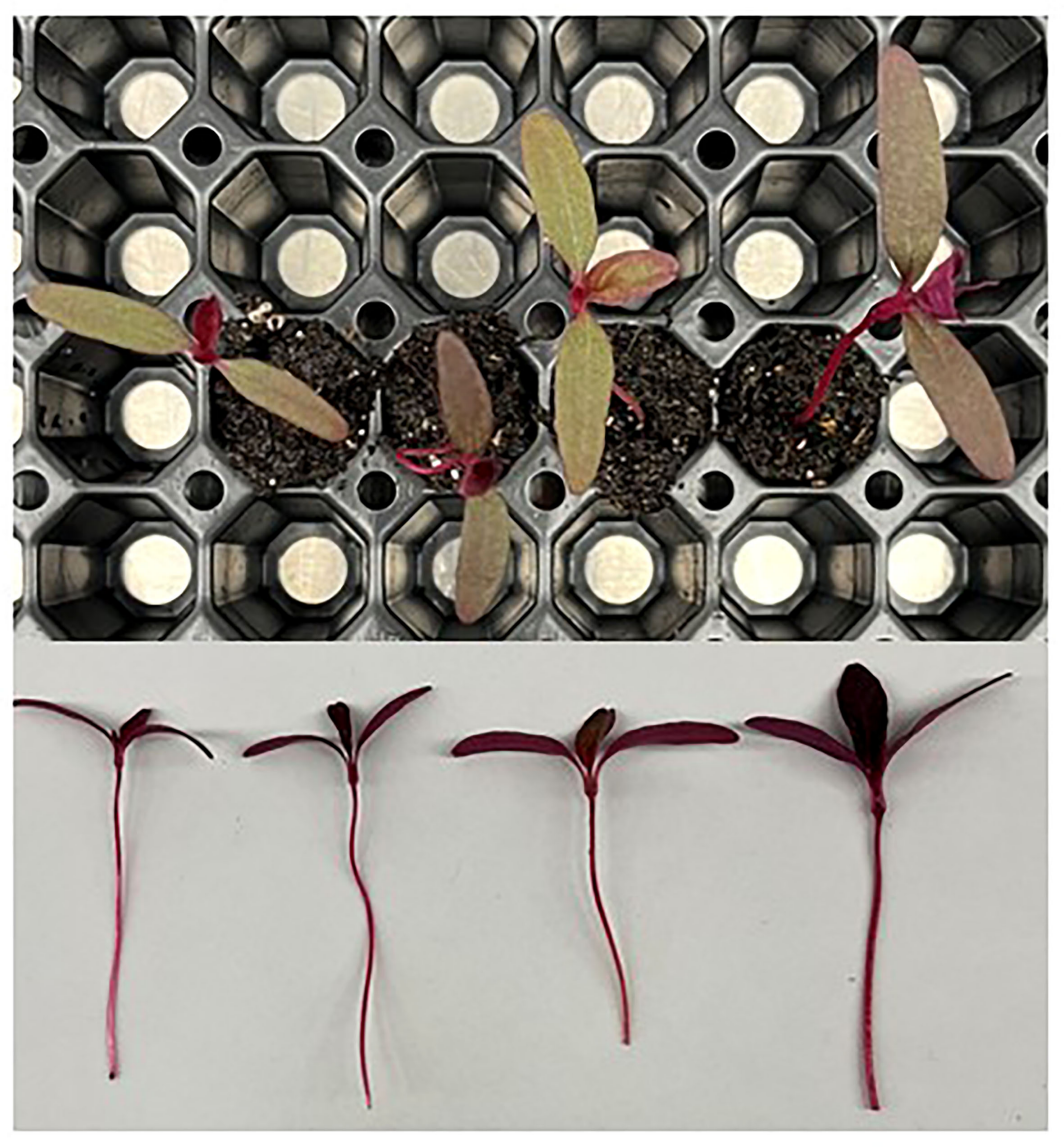
Amaranth plants grown under various lighting DLI and photoperiods. Top photo is an overhead picture, while the bottom is a side profile. From left to right, the lighting treatments are as follows (DLI/photoperiod): 14DLI/16h, 14DLI/24h, 21DLI/16h, and 21DLI/24h.

Amaranth plants were observed to be shortest in height under the 21DLI/16h treatment which was a result of the high PPFD used (**Table 2**). Both light treatments that ran for 24h produced the tallest plants regardless of DLI (**Table 2** **;** **Figure 2**). Fresh weight, a determination of yield for microgreens, was highest under the 21DLI/24h light treatment andlowest under the 14DLI/16h lighting treatment. Although the DLI was the same, both lighting treatments which utilized a 24h photoperiod produced more plant fresh biomass than did plants grown under the 16h photoperiod (**Table 2**). The energy-use-efficiency of the lights (EUEL) only is a measure of biomass accumulation normalized for the input energy of the lighting fixture. For amaranth, both 24h lighting treatments had the highest EUEL indicating that the input energy produced a higher biomass than the 16h treatment (**Table 2**). The percentage of dry matter was also highest under the 21DLI/24h light treatment while both light treatments with a low DLI had the lowest percentage of dry matter.

**Table 2:** Growth measurement summary of Amaranth cv. ‘Garnet Red’ grown under various lighting DLI and photoperiods.

Daily Light Integral (mol m^-2^ d^-1^)	Photoperiod (h)		Height (cm)	Fresh Weight (g)	EUEL (g FW MJ^-1^)	% Dry Matter
**Amaranth cv. ‘Garnet Red’**						
14		16	4.32 ± 0.16^AB^	6.47 ± 0.29^C^	0.26 ± 0.01^B^	7.12 ± 0.12^C^
		24	4.65 ± 0.19^A^	8.53 ± 0.58^B^	0.35 ± 0.02^A^	7.38 ± 0.25^BC^
21		16	3.91 ± 0.32^B^	8.90 ± 0.59^B^	0.24 ± 0.02^B^	7.92 ± 0.17^B^
		24	4.55 ± 0.09^A^	12.47 ± 0.61^A^	0.34 ± 0.02^A^	8.76 ± 0.13^A^
Daily Light Integral			0.0624	<0.0001	0.2441	0.0004
Photoperiod			0.0048	0.0001	0.0001	0.0111
Daily Light Integral*Photoperiod			0.2126	0.0628	0.5036	0.1062

Height was subsampled at five locations within the tray at the end of each of the three respective growth trials. Values presented are the means of three replicates, one from each trial ± the standard error of the means. Different letter groups (A, B, C) represent statistical differences as determined by a two-way ANOVA within each parameter at p<0.05.

**Figure 3:**
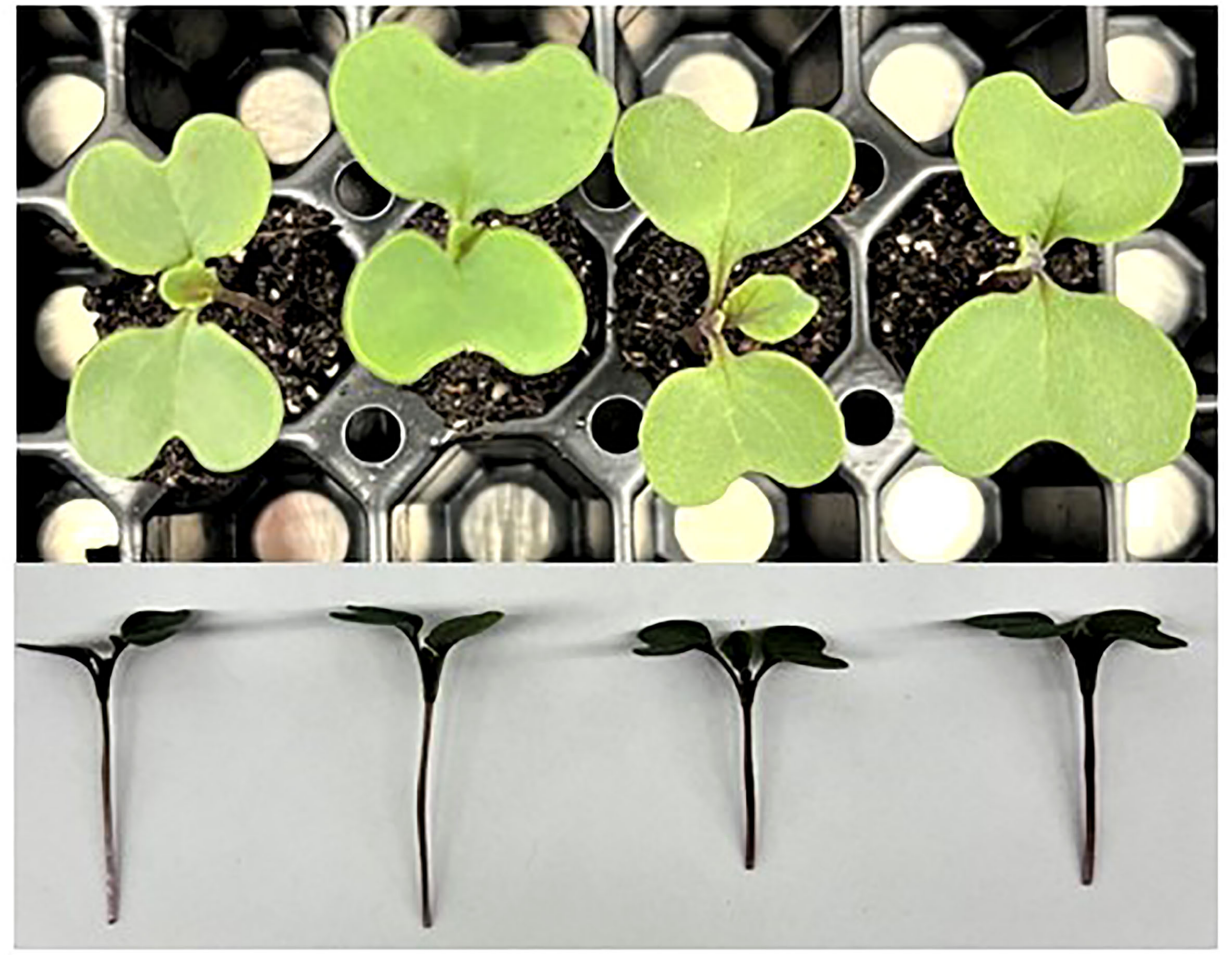
Collard greens plants grown under various lighting DLI and photoperiods. Top photo is an overhead picture, while the bottom is a side profile. From left to right, the lighting treatments are as follows (DLI/photoperiod): 14DLI/16h, 14DLI/24h, 21DLI/16h, and 21DLI/24h.

Similar to amaranth, collard greens grown under 14DLI/24h which utilized the lowest PPFD produced the tallest plants while plants grown under the highest PPFD (21DLI/16h) were the shortest (**Table 3** **;** **Figure 3**). Fresh weight was the lowest in plants grown under the 14DLI/16h treatment and the highest under the 21DLI/24h treatment (**Table 3**). Interestingly, although the DLI was lower, plants grown under the 14DLI/24h light treatment produced similar fresh weight to both treatments with high DLIs of approximately 21 mol m^-2^ d^-1^ (**Table 3**). Collard green plants grown under the 14DLI/24h treatment had the highest EUEL as more biomass was produced with the least amount of input energy (**Table 3**). Plants grown under both high DLI treatments had the lowest EUEL regardless of photoperiods. Percentage of dry matter was the highest in both 21DLI/16h and 21DLI/24h compared to treatments with low DLIs of approximately 14 mol m^-2^ d^-1^.

**Table 3:** Growth measurement summary of Collard greens cv. ‘Vates’ grown under various lighting DLI and photoperiods.

Daily Light Integral (mol m^-2^ d^-1^)	Photoperiod (h)	Height (cm)	Fresh Weight (g)	EUEL (g FW MJ^-1^)	% Dry Matter
**Collard Greens cv. ‘Vates’**					
14	16	3.44 ± 0.22^AB^	20.40 ± 1.81^B^	0.82 ± 0.07^B^	9.87 ± 0.04^B^
	24	3.88 ± 0.06^A^	23.37 ± 2.28^AB^	0.95 ± 0.09^A^	9.98 ± 0.47^B^
21	16	3.09 ± 0.22^B^	22.40 ± 2.29^AB^	0.60 ± 0.06^C^	11.95 ± 0.11^A^
	24	3.64 ± 0.22^AB^	24.70 ± 2.39^A^	0.67 ± 0.07^C^	13.23 ± 0.24^A^
Daily Light Integral		0.0888	0.0420	<0.0001	<0.0001
Photoperiod		0.0153	0.0066	0.0061	0.0425
Daily Light Integral*Photoperiod		0.7032	0.6248	0.2994	0.0757

Height was subsampled at five locations within the tray at the end of each of the three respective growth trials. Values presented are the means of three replicates, one from each trial± the standard error of the means. Different letter groups (A, B, C) represent statistical differences as determined by a two-way ANOVA within each parameter at p<0.05.

**Figure 4:**
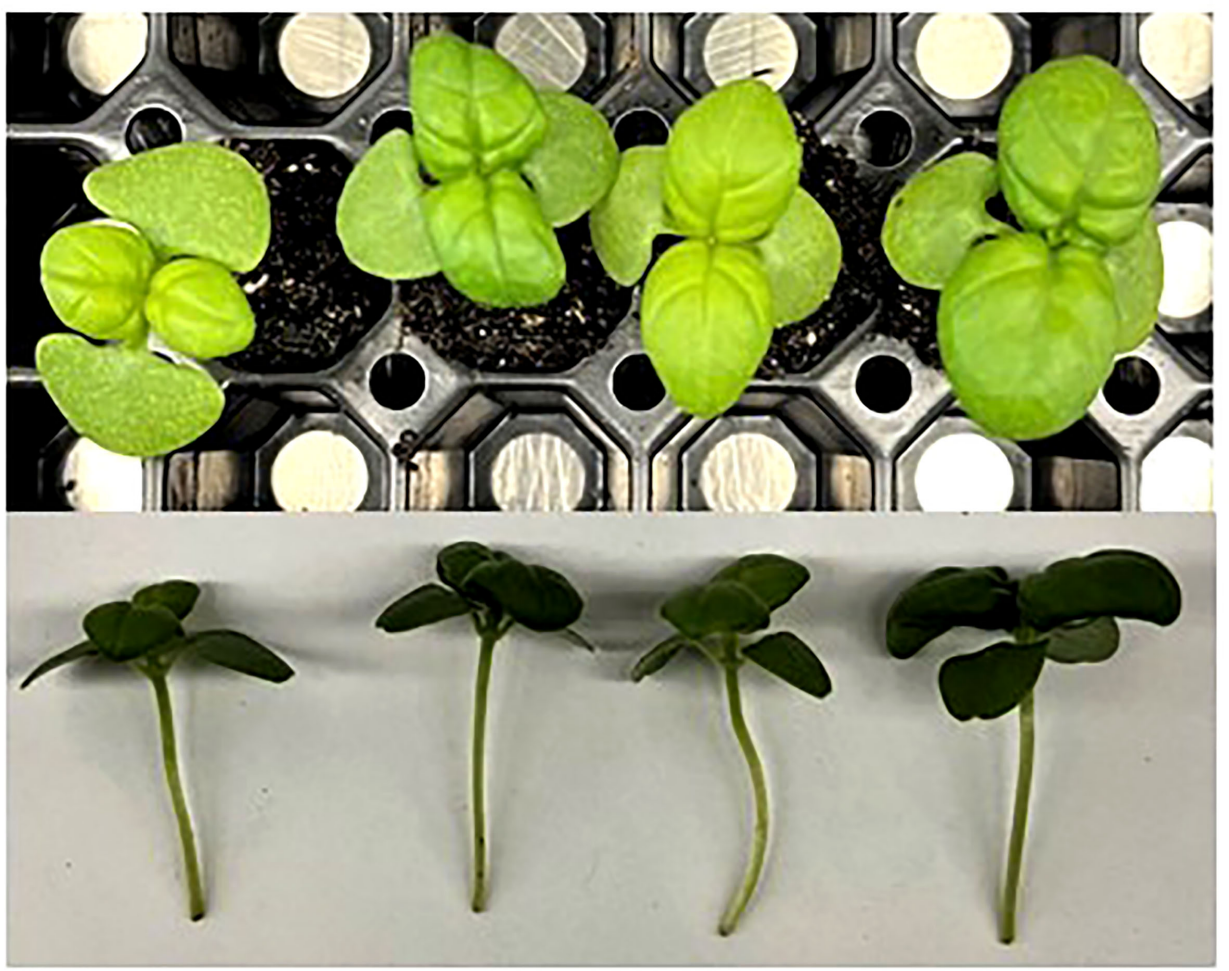
Green basil plants grown under various lighting DLI and photoperiods. Top photo is an overhead picture, while the bottom is a side profile. From left to right, the lighting treatments are as follows (DLI/photoperiod): 14DLI/16h, 14DLI/24h, 21DLI/16h, and 21DLI/24h.

Green basil plants were tallest when grown under 21DLI/24h, while those grown under 14DLI/16h were the shortest (**Table 4** **;** **Figure 4**). The same trend was noticed in fresh weight production where plants under 21DLI/24h produced the highest biomass while those under 14DLI/16h produced the least (**Table 4**). In green basil, both 24h lighting treatments had higher EUEL than did their 16h counterparts which is both a factor of increased biomass production and the lower input energy required by these treatments (**Table 4**). Notably, plants grown under the 14DLI/24h treatment produced the highest EUEL among all treatments. While the fresh weight produced was not the highest, the input energy required to produce the fresh weight was the lowest of all treatments, resulting in the highest EUEL. However, while plant height, fresh weight, and EUEL were impacted by the lighting treatments, the percentage of dry matter was similar between all treatments indicating no increase in water uptake under different light treatments (**Table 4**).

**Table 4:** Growth measurement summary of green basil cv. ‘Genovese’ grown under various lighting DLI and photoperiods.

Daily Light Integral (mol m^-2^ d^-1^)	Photoperiod (h)	Height (cm)	Fresh Weight (g)	EUEL (g FW MJ^-1^)		% Dry Matter
**Green Basil cv. ‘Genovese’**						
14	16	2.75 ± 0.12^C^	16.33 ± 1.05^C^	0.66 ± 0.04^B^	10.17 ± 1.20^A^	
	24	2.99 ± 0.13^B^	20.30 ± 0.97^B^	0.82 ± 0.04^A^	10.29 ± 0.50^A^	
21	16	2.98 ± 0.08^B^	19.30 ± 0.47^B^	0.52 ± 0.01^C^	11.73 ± 0.44^A^	
	24	3.28 ± 0.15^A^	23.37 ± 0.58^A^	0.64 ± 0.02^B^	10.93 ± 0.75^A^	
Daily Light Integral		0.0003	0.0010	0.0002		0.0378
Photoperiod		0.0003	0.0002	0.0005		0.4453
Daily Light Integral*Photoperiod		0.4284	0.9245	0.3046		0.3040

Height was subsampled at five locations within the tray at the end of each of the three respective growth trials. Values presented are the means of three replicates, one from each trial ± the standard error of the means. Different letter groups (A, B, C) represent statistical differences as determined by a two-way ANOVA within each parameter at p<0.05.

**Figure 5:**
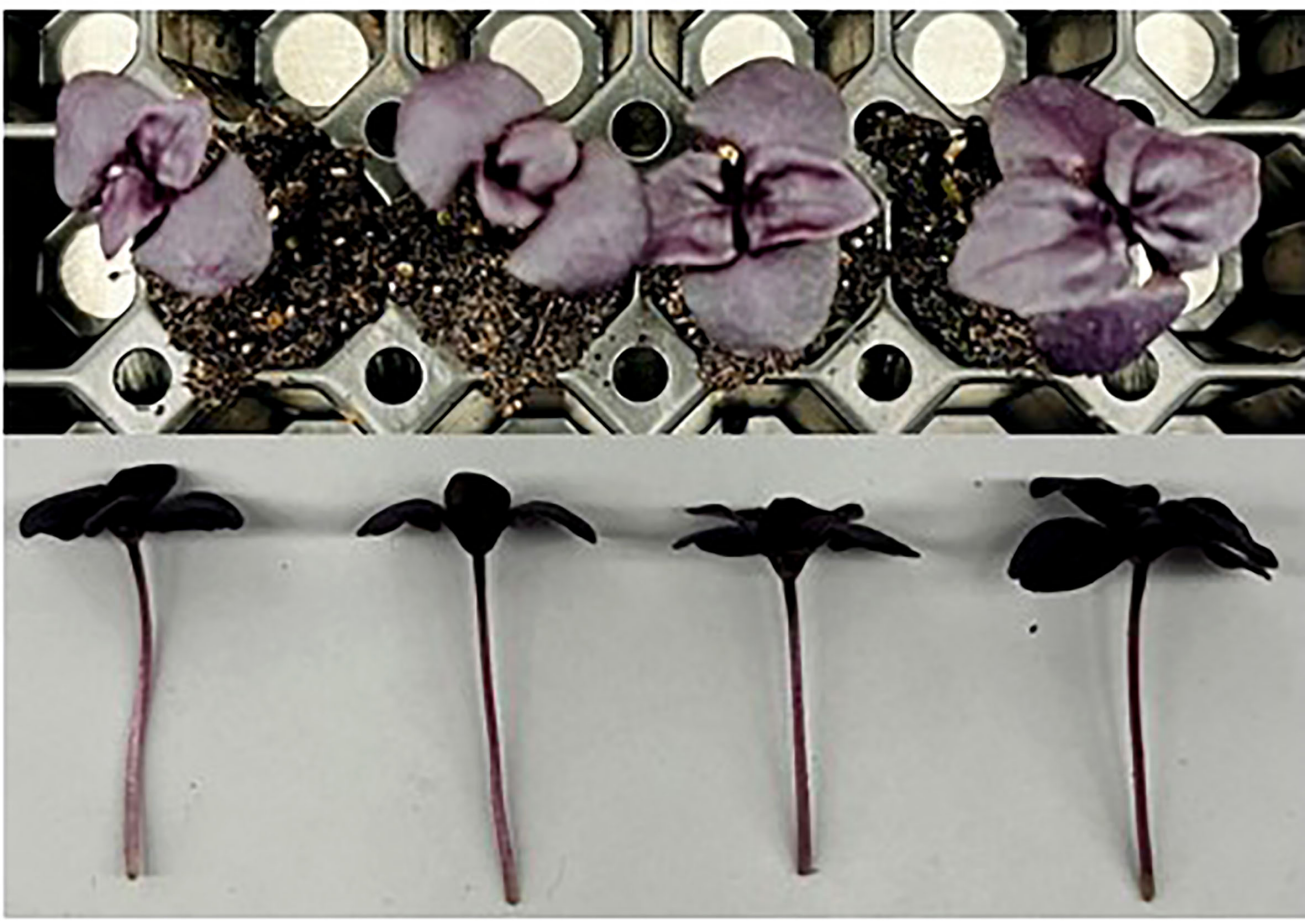
Purple basil plants grown under various lighting DLI and photoperiods. Top photo is an overhead picture, while the bottom is a side profile. From left to right, the lighting treatments are as follows (DLI/photoperiod): 14DLI/16h, 14DLI/24h, 21DLI/16h, and 21DLI/24h.

Similar to green basil, purple basil plants were shortest under 14DLI/16h and the tallest under 21DLI/24h (**Table 5** **;** **Figure 5**). Total fresh weight was observed to be the highest when plants were grown under the high DLI of approximately 21 mol m^-2^ d^-1^ and the lowest under the low DLI of approximately 14 mol m^-2^ d^-1^ regardless of photoperiods. Consistent with the green basil results, purple basil plants grown under the 14DLI/24h light treatment had the highest EUEL due to having the lowest input energy. Notably, both 24h lighting treatments had higher EUEL than the 16h treatments at the same DLI (**Table 5**). Coinciding with the results from green basil, the percentage of dry matter of purple basil was similar regardless of treatments. Interestingly, although both green and purple basils are the same species, both fresh weight and percentage of dry matter were lower in purple basil compared to green basil (**Tables 4**, **5**).

**Table 5:** Growth measurement summary of purple basil cv. ‘Red Rubin’ grown under various lighting DLI and photoperiods.

Daily Light Integral (mol m^-2^ d^-1^)	Photoperiod (h)	Height (cm)	Fresh Weight (g)	EUEL (g FW MJ^-1^)		% Dry Matter
**Purple Basil cv. ‘Red Rubin’**						
14	16	3.02 ± 0.18^B^	10.67 ± 0.50^B^	0.43 ± 0.02^BC^	7.20 ± 0.51^A^	
	24	3.17 ± 0.13^AB^	12.07 ± 0.32^B^	0.49 ± 0.01^A^	7.05 ± 0.88^A^	
21	16	3.21 ± 0.15^AB^	14.77 ± 0.67^A^	0.40 ± 0.01^C^	7.53 ± 0.58^A^	
	24	3.30 ± 0.10^A^	15.53 ± 0.71^A^	0.44 ± 0.01^B^	7.75 ± 0.77^A^	
Daily Light Integral		0.0104	0.0001	0.0330		0.0398
Photoperiod		0.0329	0.0480	0.0197		0.8678
Daily Light Integral*Photoperiod		0.5624	0.4964	0.5373		0.3966

Height was subsampled at five locations within the tray at the end of each of the three respective growth trials. Values presented are the means of three replicates, one from each trial ± the standard error of the means. Different letter groups (A, B, C) represent statistical differences as determined by a two-way ANOVA within each parameter at p<0.05.

### 3.2 Photosynthetic pigments

**Figure 6:**
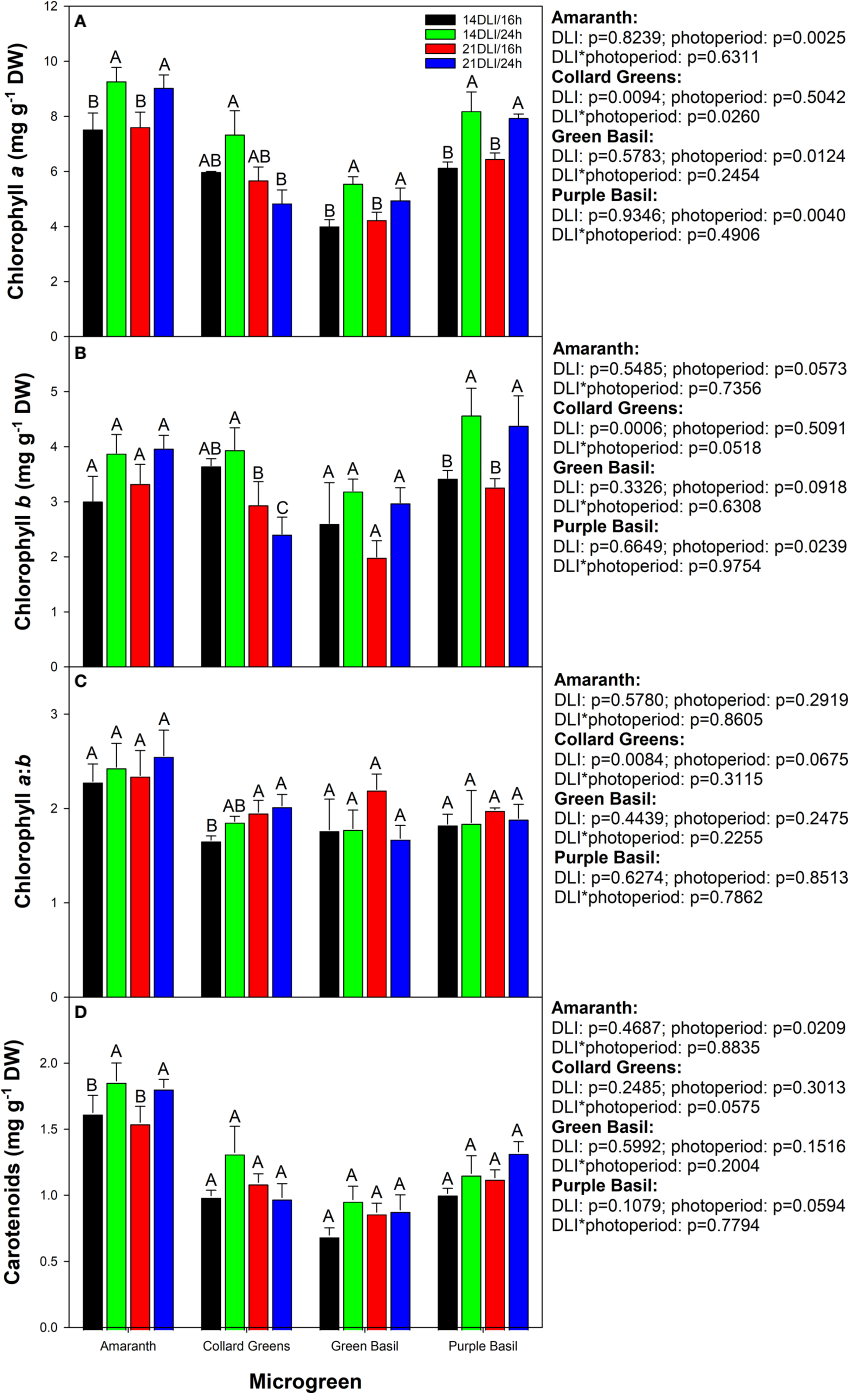
Photosynthetic pigment analysis of amaranth, collard greens, green basil, and purple basil grown under 14DLI/16h, 14DLI/24h, 21DLI/16h, and 21DLI/24h lighting treatments. Chlorophyll *a*, chlorophyll *b*, chlorophyll *a*:*b*, and carotenoids are shown in panels **(A–D)** respectively. Values presented are the means of two subsamples from each of the three replicates ± the standard error of the means. Different letter groups (A–C) represent statistical differences with microgreen type and panel as determined by a two-way ANOVA within each parameter at p<0.05. P-values are shown to the right of their respective panels for each microgreen.

Chlorophyll, as well as carotenoids, play a role in light harvesting for photosynthesis. However, in microgreens, they also provide vibrant green, yellow, and red colours which are sought after by chefs. In amaranth, green basil, and purple basil, growth under both 24h lighting treatments produced the highest chlorophyll *a* content (**Figure 6A**). In collard greens, chlorophyll *a* was the highest in the 14DLI/24h treatment but observed to be the lowest in the 21DLI/24h treatment. Chlorophyll *b* was not affected by light treatments in both amaranth and green basil (**Figure 6B**). In collard greens, similar to chlorophyll *a*, chlorophyll *b* was observed to be the highest in the 14DLI/24h treatments and the lowest in the 21DLI/24h treatment. In addition, both high DLI treatments were observed to have lower chlorophyll *b* content than the 14DLI/24h light treatments. In purple basil, both 24h light treatments had higher chlorophyll *b* content than did the 16h treatments (**Figure 6B**). The chlorophyll *a*:*b* was observed to be similar between light treatments in amaranth and both basil microgreens (**Figure 6C**). However, in collard greens, the chlorophyll *a*:*b* was the lowest in the 14DLI/16h treatment and the highest in both high DLI treatments. Carotenoids were the highest in both 24h lighting treatments in amaranth but the other three microgreens were unaffected by light treatments (**Figure 6D**).

### 3.3 Antioxidants

**Figure 7:**
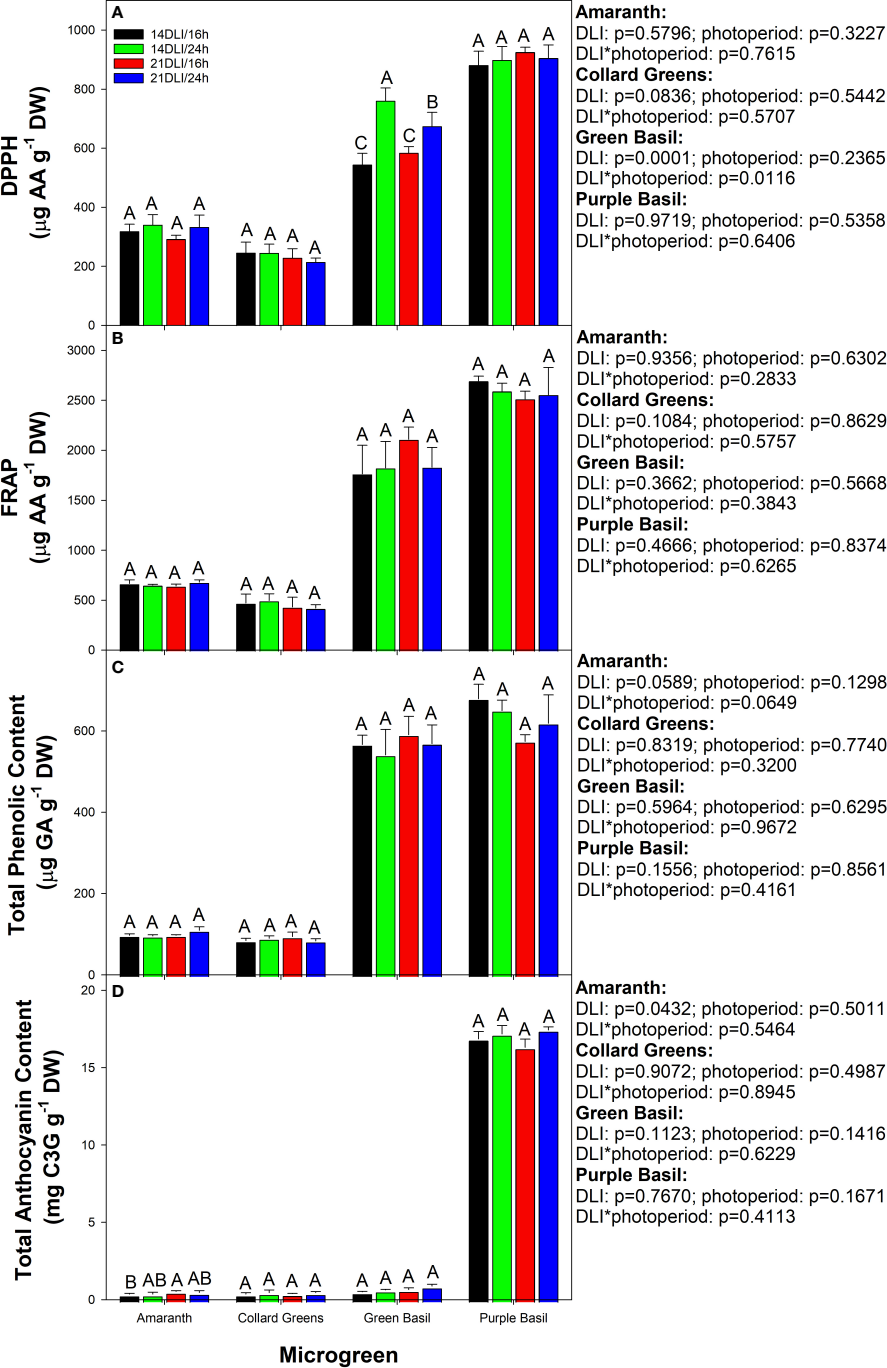
Antioxidant activities in microgreens as measured by 2,2-diphenyl-1-picrylhydrazyl (DPPH; Panel **A**), ferric reducing antioxidant power (FRAP; Panel **B**), total phenolic content (Panel **C**), and total anthocyanin content (Panel **D**) of all microgreens grown under 14DLI/16h, 14DLI/24h, 21DLI/16h, and 21DLI/24h lighting treatments. Values presented are the means of two subsamples from each of the three replicates ± the standard error of the means. Different letter groups (A–C) represent statistical differences with microgreen type and panel as determined by a two-way ANOVA within each parameter at p<0.05. P-values are shown to the right of the panel.

Microgreens are prized for their antioxidant and nutrient densities in comparison to their mature counterparts. Here we see that the antioxidant activity as measured by 2,2-diphenyl-1-picrylhydrazyl (DPPH) assay was increased in both 24h lighting treatments compared to their 16h counterparts at the same DLI in green basil (**Figure 7A** **)**. Furthermore, plants grown under the 14DLI/24h light treatment had the highest DPPH activity of all light treatments in green basil. DPPH activity was unaffected by light treatments in all other microgreens. Similarly, ferric reducing antioxidant power (FRAP) was observed to be unaffected by light treatments in all microgreens (**Figure 7B** **)**. Phenolics, which can provide resistance against various biotic and abiotic stress conditions the plant is under, were unaffected by the different light treatments (**Figure 7C** **)**. In amaranth, the anthocyanin content was observed to be the highest under the 21DLI/16h treatment and the lowest under the 14DLI/16h treatment (**Figure 7D** **)**. Anthocyanin content was unaffected by light treatments in all other microgreens (**Figure 7D** **)**. A trend which can be observed is that there is higher antioxidant activity and phenolic and anthocyanin content in both basil microgreens in comparison to amaranth and collard greens. Notably, purple basil tends to have the highest antioxidant capacity as well as phenolic and anthocyanin concentrations, which is in part a cause of its deep purple colouration.

## 4 Discussion

### 4.1 Continuous lighting and microgreen growth

Both PPFD and photoperiod are known to impact plant morphology. Growth under low PPFD will increase leaf area in order to maximize the area capable of intercepting incoming light (Palmer and van Iersel, 2020). Conversely, high PPFD will lead to a reduction in specific leaf area (i.e., smaller, thicker leaves) to protect the plant from high irradiance levels in order to minimize damage due to excessive light (Matos et al., 2009; Fan et al., 2013). Extended photoperiods including CL have led to smaller leaf area in tomatoes (Velez-Ramirez et al., 2014), which is a similar attribute seen in plants grown under high PPFD in order to avert damage due to excess light.

In this study, with the exception of collard greens, we observed that plants grown under the 21DLI/24h treatment were the tallest (**Tables 2** **–** **5**). Furthermore, and again with the exception of collard greens, leaf area was visually larger when plants were grown under the 21DLI/24h treatment (**Figures 2** **–** **5**). Increases in both plant height and leaf area are traits typically associated with growth in low light environments (Poorter et al., 2019). However, there is an interplay between leaf expansion due to low light and photosynthesis driven by adequate PPFD. Compared to the 21DLI/16h treatment, the 21DLI/24h treatment used lower PPFD which enabled greater leaf expansion. In turn, the larger leaf expansion allowed for greater light interception and thus higher overall plant photosynthesis leading to increased biomass. This notation is supported by an increased EUEL of plants grown under the 24h photoperiods compared to their respective 16h counterparts. It should also be noted that all 24h lighting treatments in amaranth, green basil, and purple basil had elevated levels of chlorophyll (**Figure 5**). Being the major photosynthetic pigment, a strong correlation can be drawn between chlorophyll content and increased photosynthesis leading to greater biomass accumulation (Buttery and Buzzell, 1977). It has been observed that amaranth growth under a 20h photoperiod had the highest fresh biomass while also having increased levels of chlorophyll *a* (Meas et al., 2020). Interestingly, collard greens do not show the same obvious enhancement in leaf size or biomass accumulation under CL. This also coincided with similar or lower chlorophyll content when comparing the 24h treatments to the 16h treatments (**Figure 6**). Photosynthetic pigment concentration was observed to increase in basil, rocket, and chicory plants grown under a similar 24h light treatment as used in this manuscript (Pennisi et al., 2020). Similar to the results in amaranth and both basil cultivars, Weaver and van Iersel (2020) observed that lettuce had increased leaf area and dry biomass when the photoperiod was extended, but the DLI stayed the same. In contrast to our study, Pennisi et al. (2020) did not observe an increase in fresh biomass accumulation in basil when grown under a 24h photoperiod with a DLI of 21.6 mol m^-2^ d^-1^. The difference in observations could be a result of differences in plant age. In Pennisi et al. (2020) the 24h treatment began when the plants were 21 days old whereas in our study, the 24h treatment began when the plants were only 5 days old. In this way, no mutual shading had occurred in our study (due to the small size of the plants) allowing maximum photon capture by the plant which resulted in greater biomass accumulation. Accordingly, an increase in photon capture and biomass accumulation would be negated when the canopy is fully matured and vegetation is dense. Since microgreens are typically harvested before or at the first true leaf stage, this competitive advantage when plants are young would lead to larger plants as observed in this study, or reduced production times as the plants would reach the desired size more quickly when grown under a long photoperiod with low PPFD (i.e., 21DLI/24h) as opposed to a shorter photoperiod and higher PPFD (i.e., 21DLI/16h).

In contrast to traditional morphological responses to CL, generally speaking, the microgreens in this study grown under 24h lighting had visually larger leaves than those grown under 16h. Reduced leaf size due to CL has been seen in tomatoes (Velez-Ramirez et al., 2014; Pham et al., 2019). However, microgreens grown under CL have been shown to have increased leaf area (**Figures 2** **–** **4**; Shivaeva et al., 2022b). This may indicate that microgreens have a higher PPFD threshold before morphological adaptation occurs to reduce light capture. In a model analysis on tomatoes, a theoretical increase in yield of 22-26% (depending on PPFD) has been predicted when grown under CL if injury could be averted (Velez-Ramirez et al., 2012). In this study, yield increases were 92.7%, 21.1%, 43.1%, and 45.5% for amaranth, collard greens, green basil, and purple basil, respectively when the photoperiod was extended from 16h to 24h at the same PPFD (i.e., comparing 14DLI/16h and 21DLI/24h). Our results show equal or higher yield increases compared to the theoretical model analysis for tomatoes. This difference in result is likely two-fold. Firstly, as opposed to the complex canopy of tomatoes, microgreens in this study had very little mutual shading during their production and consequently all of the leaf area was able to absorb light, maximizing photosynthesis. Secondly, unlike tomatoes which produce fruit, all of the above ground biomass of the microgreen is edible and therefore all of the assimilated carbon contributes to yield increase. Therefore, due to the simplicity of microgreens, a greater yield return is observed during CL when compared to the more complex crop of tomatoes.

### 4.2 Maintaining nutritional content during CL production

Microgreens are prized for their nutrient profile, vibrant colours, and flavour which often allow chefs to add new dimensions to their dishes. Microgreens are also increasingly becoming popular as an everyday leafy green due to their high antioxidant content making them a functional food (Xiao et al., 2012; Kyriacou et al., 2019). Here we see that, generally speaking, antioxidant, phenolic, and anthocyanin content remained similar or increased when microgreens were grown under a high DLI or an extended photoperiod. Coupling this with the reduced electricity cost of microgreens grown under 24h lighting, utilizing CL for microgreen production can produce plants at a reduced cost without sacrificing nutrient density.

Both amaranth and collard greens showed improved dry matter content under the high DLI treatment regardless of photoperiods (**Tables 2** and **3**). Additionally, amaranth was observed to have improved dry matter content under the 24h photoperiod compared to the 16h photoperiod at the high DLI. Since DPPH and FRAP activities (**Figures 7A, B**) as well as phenolic (**Figure 7C** **)** and anthocyanin (**Figure 7D**) content were expressed on a dry weight basis and were similar, it then stands to reason that under the high DLI treatment, both amaranth and collard greens have improved antioxidant, phenolic, and anthocyanin content due to their higher dry matter content. Amaranth has also been shown to have increased anthocyanin production when grown under 280 µmol m^-2^ s^-1^ compared to lower PPFD values (Meas et al., 2020). What’s more is that for amaranth, growth under CL at the high DLI also improved overall nutrient content compared to the 21DLI/16h treatment.

Both an increase in DLI and photoperiod extension are known to impact the secondary metabolite concentrations within plants (Samuoliene et al., 2013). Increasing the PPFD during growth and/or extending the photoperiod can cause an abiotic stress response within plants as additional light is being provided, and, in this study, the plant is under CL (Demmig-Adams and Adams, 1992; Sairam et al., 2001; Brazaityte et al., 2015; Haque et al., 2015; Gharibi et al., 2016; Szymańska et al., 2017). The stress response is characterized by an increase in reactive oxygen species (ROS) and free radicals which can be detrimental to plant health if not properly addressed, causing damage to the photosynthetic machinery (Arora et al., 2002; Pospisil et al., 2016; Huang et al., 2019). To counter the increase in free radicals, an increase in antioxidants is needed. Here, we see that under the high DLI conditions for collard greens and under high DLI and CL in amaranth, an increase in DPPH and FRAP activities was observed (**Figures 7A, B**). This, coupled with increased phenolic and anthocyanin levels, helps to reduce oxidative stress in the plant by channeling extra light energy away from the light harvesting complex and removing free radicals (Huang et al., 2019). Similar responses have been noted in tomatoes (Haque et al., 2015), lettuces (Zha et al., 2019), mung beans (Kumar et al., 2022), and *Brassicaceae* microgreens (Shibaeva et al., 2022b).

Notably, the increase in DPPH, FRAP, phenolics, and anthocyanins was only observed to occur in amaranth and collard greens and was absent in both basil microgreens with the exception of DPPH in green basil which was higher in the 24h treatments than the 16h treatments. However, green and purple basil had higher levels of all secondary metabolites compared to amaranth and collard greens (**Figures 7** **)**. Basil is known for its incredible aroma and antioxidant concentration (Ciriello et al., 2021). Due to its already high secondary metabolic concentrations, further enhancement due to any hormetic effect of increased PPFD/DLI or photoperiod was not observed. Sutulienė et al. (2022) also observed no increase in FRAP and DPPH activity as well as total phenolic and anthocyanin content in basil when the PPFD was increased from 150 to 250 µmol m^-2^ s^-1^. Samuoliene et al. (2013) noted the impact of PPFD is species-specific with respect to secondary metabolite concentrations. This suggests that further studies need to be done to identify species-specific secondary metabolite responses to various lighting conditions.

While increases in antioxidants, phenolics, and anthocyanins can have a beneficial response in plants under environment stressors, they can also be advantageous to humans during consumption. Similar to their radical-scavenging abilities in plants, these compounds have been shown to reduce the risk of cardiovascular disease, diabetes, cancer, and even mitigate age-related diseases in humans (Lobo et al., 2010; Engwa, 2018). Therefore, growing microgreens under high DLI and CL can produce a hormetic effect in which the plant responds to the stress of high light and/or a long photoperiod by increasing the production of important secondary metabolites which also happen to be health promoting compounds for humans.

### 4.3 Continuous lighting can improve yield and lower electricity cost

Since microgreens are sold on a fresh weight basis, the main goal of plant factories producing microgreens is to increase fresh biomass while minimizing inputs and maintaining nutritional content. A traditional way to increase biomass is to increase the DLI either through increased PPFD or extended photoperiods (Samuoliene et al., 2013; Meas et al., 2020). In this study, all microgreens tested, with the exception of collard greens, showed an increase in fresh weight when the DLI increased from approximately 14 mol m^-2^ d^-1^ to approximately 21 mol m^-2^ d^-1^ when the photoperiod was 16h. In general, as the DLI increases, one would expect biomass to increase as well (up to a saturation point) because more light means more photo-assimilation (Poorter et al., 2019). The increase in biomass seen here with an increase in DLI is in-line with results from previous works with other microgreen species including broccoli, arugula, mizuna, radish, tatsoi, and red pak choi (Samuoliene et al., 2013; Shibaeva et al., 2022b). However, in all microgreens studied, an increase in production due to increased DLI during a 16h photoperiod was associated with the same or increased electricity cost due to the additional light needed (**Table 6**). In this way, resource-use-efficiency actually decreased as DLI increased.

**Table 6:** Electricity cost from LED lighting only of microgreens under various DLI and photoperiods.

Daily Light Integral (mol m^-2^ d^-1^)	Photoperiod (h)	Electricity Cost ($ g^-1^ FW)			
		Amaranth	Collard Greens	Green Basil	Purple Basil
14	16	0.41 ± 0.01^A^	0.13 ± 0.02^B^	0.65 ± 0.01^B^	0.44 ± 0.03^AB^
	24	0.27 ± 0.02^B^	0.10 ± 0.01^C^	0.60 ± 0.01^C^	0.34 ± 0.02^C^
21	16	0.45 ± 0.02^A^	0.18 ± 0.02^A^	0.76 ± 0.02^A^	0.48 ± 0.04^A^
	24	0.28 ± 0.02^B^	0.14 ± 0.01^B^	0.66 ± 0.02^B^	0.39 ± 0.03^B^
Daily Light Integral		0.2999	0.0001	0.0003	0.0085
Photoperiod		<0.0001	0.0005	<0.0001	0.0002
Daily Light Integral*Photoperiod		0.5666	0.2023	0.1729	0.1824

All electrical prices are for Ontario, Canada and were obtained from the Independent Electricity System Operator (IESO, 2022) for the given production periods (**Supplementary Table 1**). All prices are in Canadian dollars. Electricity usage was calculated using the Flexstar 645W with appropriate dimming capabilities to reach the desired light intensities (**Table 1**). Electricity costs are calculated by using the total electricity costs related to lighting for each production period (i.e., replicate) and dividing that by the actually fresh biomass produced in said production period. Values presented are the means of three growth trials, each representing one replicate ± the standard error of the means. Different letter groups (A, B, C) represent statistical differences as determined by a two-way ANOVA within each parameter at p<0.05.

In this study, the continuous 24h lighting uses low intensity light throughout the production period of the plant. In this way, the plant is under constant illumination and is continuously photosynthesizing; thereby negating dark respiration and therefore, no loss of carbon occurs during the night. In fact, most plants are observed to have elevated leaf carbohydrate levels when grown under CL when nighttime light intensities are above the light compensation point (Globig et al., 1997; Matsuda et al., 2014; Pham et al., 2019). In microgreens which do not export fixed carbon to a growing fruit, CL-injury did not occur in any of the four microgreens which were studied. The extended photoperiod translated to an increase in biomass due to the accumulation of carbohydrates in the leaves – increasing resource-use-efficiency of the production system (**Table 6**). In fact, EUEL was increased by 10-42% and electricity cost of the light fixtures was decreased by 8-38% depending on DLIs and microgreens, when transitioning from a 16h to a 24h photoperiod.

Due to the nature of plant factories being inside buildings, all lighting requirements needed by the plant must be achieved through sole-source lighting such as LEDs. Electricity is then one of the largest input cost components for plant factories (Kozai and Niu, 2019; van Delden et al., 2021). CL can reduce the number of lighting fixtures needed (compared to a similar DLI at a 16h photoperiod) which will reduce the initial fixture cost – often a large barrier to entry into controlled environment agriculture, specifically plant factories (Hao et al., 2018). Furthermore, CL can reduce electrical costs *via* the use of lower nighttime electricity prices in regions of the world which use TOUP (**Supplementary Table 1**). While the data provided in **Table 6** is for Ontario, Canada, other regions of the world such as some US states, 17 European nations, and South Korea use time-of-use electricity pricing, so this concept would also provide a good potential to reduce electricity costs in those regions (IRENA, 2019; IESO, 2022). In regions which do not utilize TOUP, the reduction in initial fixture cost due to the lower PPFD used during CL as well as the reduced need for heat and humidity dissipation would still provide growers with financial gains (Goto, 2012; Kozai and Niu, 2016; Kozai et al., 2016; Graamans et al., 2018). Plant factories typically require extensive air conditioning in order to maintain proper temperature and humidity for plants; mostly to overcome heat generated by the LED fixtures (Wang et al., 2014; Graamans et al., 2018) and to remove the moisture from plant transpiration. The use of CL not only reduces the overall amount of fixtures, but allows the grower to reduce the PPFD by 33% at the same DLI (**Table 1**). Together, both the reduction in overall fixtures and lower PPFD used means less heat and moisture will be produced within the plant factory which in turn translates to less need for energy consuming air conditioning. The use of CL and subsequent reduction in fixtures needed, heat emittance, and moisture generation can also be useful for space travel as power consumption and heat loss can be large challenges in self-supporting food production (Stutte, 2015).

In this study, at the same DLI, amaranth and green basil produced higher fresh biomass when grown under CL when compared to the 16h lighting treatments (**Tables 2**, **4**). Even in collard greens and purple basil, the use of reduced electrical prices during the night lowered electricity cost in 24h treatments making them more cost effective than their 16h counterparts (**Table 6**). The microgreens studied here join a growing list of plants which can tolerate CL including other microgreen species (Shibaeva et al., 2022b), lettuces (Ohtake et al., 2018), cucumbers (Lanoue et al., 2021b), peppers (Lanoue et al., 2022), and tomatoes (Haque et al., 2017; Lanoue et al., 2019).

## 5 Conclusion

Plant factories require the use of sole-source lighting with intensive energy input. It usually requires high capital investment due to the high costs of LED and HVAC equipment. CL has been studied in many plant systems and represents a potential strategy to lower fixture needs and reduce PPFD during prolonged photoperiods; resulting in reduced electricity costs. Here we show that four microgreens, amaranth, collard greens, green basil, and purple basil have increased fresh biomass accumulation and/or reduced electricity costs when grown under CL regardless of DLIs. Furthermore, the use of high DLI in collard greens and high DLI and CL in amaranth increased DPPH and FRAP activities as well as phenolic and anthocyanin content. Green basil and purple basil maintained their secondary metabolite concentrations while still having reduced electricity costs when grown under CL. In this way, the use of CL for microgreen production can improve energy efficiency while maintaining or increasing antioxidants, phenolics, and anthocyanins making it a more sustainable lighting strategy than high intensity short photoperiod lighting.

## Data availability statement

The original contributions presented in the study are included in the article/**Supplementary Material**. Further inquiries can be directed to the corresponding author.

## Author contributions

JL and XH were involved in the conceptualization, methodology development, and writing. JL, XH, and CL edited the manuscript. JL SS and CL were involved in data curation and day-to-day upkeep of the experiment. JL performed data analysis. XH was responsible for funding acquisition. All authors have read and agreed to the submitted manuscript.

## Funding

The project is funded by the Foundation Science Program of Agriculture and Agri-Food Canada to XH (J-002228.001.04).

## Conflict of interest

The authors declare that the research was conducted in the absence of any commercial or financial relationships that could be construed as a potential conflict of interest.

## Publisher’s note

All claims expressed in this article are solely those of the authors and do not necessarily represent those of their affiliated organizations, or those of the publisher, the editors and the reviewers. Any product that may be evaluated in this article, or claim that may be made by its manufacturer, is not guaranteed or endorsed by the publisher.
